# Evaluation of Ki-67 expression levels in esophageal squamous cell carcinoma using dual-energy CT quantitative parameters

**DOI:** 10.3389/fonc.2025.1561256

**Published:** 2025-08-07

**Authors:** Jing Sun, Yong-ying Huang, Tao Lu, Xing-fa Chen, Hui-ping Ruan, De-chun Zheng

**Affiliations:** ^1^ Department of Radiology, Clinical Oncology School of Fujian Medical University and Fujian Cancer Hospital (Fujian Branch of Fudan University Affiliated Cancer Hospital), Fuzhou, Fujian, China; ^2^ Department of Epidemiology, Clinical Oncology School of Fujian Medical University and Fujian Cancer Hospital (Fujian Branch of Fudan University Affiliated Cancer Hospital), Fuzhou, Fujian, China

**Keywords:** quantitative parameters, Ki-67 expression, dual-energy CT (DECT), esophageal squamous cell carcinoma, nomogram

## Abstract

**Aim:**

This study investigates the use of dual-energy computed tomography (DECT) quantitative parameters to assess Ki-67 expression levels in esophageal squamous cell carcinoma (ESCC).

**Methods:**

A total of 57 ESCC patients who underwent dual-phase DECT scans were included. Key parameters measured were iodine concentration (IC), water concentration (WC), normalized iodine concentration (NIC), spectral Hounsfield unit curve slope (λHu), and effective atomic number (Zeff). Univariate and multivariate analyses identified factors associated with Ki-67 expression levels.

**Results:**

Results showed that high Ki-67 expression correlated with significantly higher Zeff and IC values in the venous phase (VP) (P = 0.047 and P = 0.049, respectively; AUC = 0.67 for both), and lower WC in the arterial phase (AP) (P = 0.021; AUC = 0.70). Multivariate analysis revealed that IC in VP and WC in AP were independent predictors of Ki-67 overexpression. The combination of these two parameters yielded an AUC of 0.78 for predicting Ki-67 overexpression, with 68.3% sensitivity, 87.5% specificity, and 73.7% accuracy.

**Conclusions:**

DECT parameters demonstrate potential for non-invasive Ki-67 status detection in ESCC, offering valuable insights for clinical diagnosis and prognosis.

## Introduction

Esophageal cancer (EC) is one of the most prevalent malignancies globally, ranking seventh in terms of incidence and sixth in terms of mortality (1), with approximately 604,000 new cases and 544,000 deaths in 2020 (1, 2). Esophageal squamous cell carcinoma (ESCC) is the most common histological subtype of EC (3–5). Although the global incidence of ESCC has generally declined, challenges remain due to its insidious onset and the absence of typical symptoms in the early stages. As a result, most patients are diagnosed at advanced stages, which complicates treatment and leads to poorer prognosis (6).

Ki-67 is a nuclear protein widely used as a marker for active cellular proliferation (7). It is expressed throughout the major phases of the cell cycle (G1, S, G2, and M phases) while remains inactive during the resting phase (G0) (8). Therefore, the presence of Ki-67 reflects the proliferative activity of cells. Elevated Ki-67 expression was significantly associated with poorer overall survival and disease-free survival in patients with ESCC (9, 10). Currently, in clinical practice, Ki-67 detection is primarily performed using immunohistochemistry (IHC), wherein antibodies against Ki-67 label its expression in tissue sections (11). This technique cannot monitor dynamic Ki-67 expression in real time, and due to tumor heterogeneity, small tissue samples may not accurately reflect overall Ki-67 expression, limiting its clinical applicability. Consequently, there is a clinical need for a reliable and non-invasive approach to assess Ki-67 expression in patients with ESCC.

Recent advancements in DECT imaging have enabled both quantitative and qualitative analysis of tissue, thereby introducing a novel dimension to CT imaging (12, 13). These images include substance-specific iodine concentration (IC) images, virtual non-contrast images, effective atomic number (Zeff) images, and energy-specific virtual monoenergetic images (VMIs). Multiple quantitative parameters derived from dual-energy computed tomography (DECT), such as normalized iodine concentration (NIC), spectral Hounsfield unit curve slope (λHu), and effective atomic number (Zeff), have been increasingly applied to predict Ki-67 expression in various tumors (12, 14–16). DECT imaging may provide potential value in the preoperative assessment of Ki-67 expression levels in ESCC.

This study aimed to explore the potential application of DECT-derived quantitative parameters in distinguishing Ki-67 status in ESCC.

## Material and methods

### Participants

This study was a retrospective single-center study conducted at hospital. The study protocol was approved by the Institutional Review Board, and the requirement for written informed consent was waived. The procedures performed in this study was in accordance with the Declaration of Helsinki. Between October 2019 and December 2020, 72 patients were diagnosed with ESCC through endoscopic biopsy, and underwent DECT scans and received surgical treatment within two weeks of diagnosis. Patients’ demographic data and histopathological diagnoses including Ki-67 were extracted from the hospital’s electronic medical records. Of these, 15 patients were excluded: 2 cases with poor image quality or unclear lesions, and 13 cases lacking Ki-67 index immunohistochemical results. There were 57 patients included in the final statistical analysis. encompassing clinical information and pathological data such as sex, age, tumor location, pathological T stage, lymph node involvement, and differentiation grade. The Ki-67 index was assessed to categorize tumors into low and high proliferative groups using a 50% threshold (17, 18).

### DECT image acquisition

All examinations were performed using a Revolution CT scanner (GE Healthcare, Milwaukee, USA) in DECT acquisition mode. The acquisition parameters included tube voltage (80–140 kV), tube current (355 mA), field of view (500 × 500 mm), image matrix (512 × 512), rotation speed (0.8 s/rotation), and slice thickness/gap (1.25/1.25 mm, reconstructed slice thickness 1.25 mm). Patients were positioned in the supine position, and chest dual-energy plain and dual-phase enhanced scans were sequentially obtained in the cranio-caudal direction. A non-ionic contrast agent (ioversol, 320 mgI/mL, Hengrui Medicine, China) was administered at a rate of 3.0 mL/s and a total dose of 1.5 mL/kg body weight. Contrast-enhanced images were acquired at 30 seconds and 65 seconds post-injection, representing the arterial phase (AP) and venous phase (VP), respectively.

### DECT image postprocessing

DECT images were uploaded to a commercial workstation (Advantage Workstation 4.6, GE Healthcare) and analyzed using GSI Viewer software (version 2.0, GE Healthcare). Two radiologists, with 6 and 10 years of experience in esophageal CT diagnostics, performed standardized measurements and analyses using a blinded and randomized approach. The standardized region of interest (ROI) measurement approach involved selecting a single axial slice that displayed the tumor’s maximum cross-sectional area and quantitatively measuring this area, along with the adjacent upper and lower planes. The average of these three measurements was used as the final data value. Three circular ROIs were placed in the solid area of the tumor on three consecutive slices, avoiding necrotic, vascular, calcified, and cystic regions. The size, shape, and location of the two-phase ROIs were kept consistent. On the same slice, ROIs of identical size were copied to obtain the normalized iodine concentration (NIC) for each phase. The slope (λHU) of the spectral curve was calculated as the CT attenuation value: λHU = (CT40keV - CT70keV)/30, where CT40 keV and CT70 keV represented tumor attenuation in 40 keV and 70 keV single-energy images, respectively. The final quantitative index included NIC, λHU, IC, WC, and Zeff for both the AP and VP.

### Statistical analyses

Statistical analyses were conducted using IBM SPSS Statistics version 26.0 and MedCalc Statistics Software version 20.0.14. Continuous variables with a normal distribution were expressed as mean ± standard deviation, while non-normal distribution continuous variables were presented as median with range. Categorical variables were reported as counts and percentages. Between-group comparisons of continuous data were performed using t-tests or Mann-Whitney U tests, depending on the distribution, while categorical data were compared using chi-square tests or Fisher’s exact tests. The Ki-67 index was categorized into low and high expression groups by median. Univariate and multivariate logistic regression analyses were conducted to identify risk factors for high Ki-67 expression. All variables with p-values < 0.1 in the univariate analysis were considered eligible for inclusion in the multivariate analysis. Multicollinearity was tested using the variance inflation factor (VIF). A VIF > 5 indicates the presence of multicollinearity. Backward stepwise selection method was applied in variable selection. The diagnostic performance of individual and combined parameters was assessed by calculating the area under the receiver operating characteristic curve (AUC). Optimal cutoff values were determined based on Youden’s index. A two-tailed p-value < 0.05 was considered statistically significant in all statistical tests.

## Results

### Patient characteristics

This study enrolled 57 patients with ESCC, consisting of 46 males and 11 females. The average age of the patients was 61.8 ± 8.98 years, with a range of 43 to 79 years. The lesions were located at the middle segment of esophagus in 24 cases, the upper segment in 4 cases, and the lower segment in 29 cases. Among the patients, 13 had highly differentiated tumors, while 44 had moderately or poorly differentiated tumors. Referring to the pathological TNM staging of ESCC (AJCC 8th edition), 13 patients were classified as stage pT1, 9 patients as stage pT2, 32 patients as stage pT3, and 3 patients as stage pT4. Regarding regional lymph node metastasis, 32 patients had no metastasis, 11 patients had metastasis involving fewer than 3 nodes (pN1), 6 patients had metastasis involving 3 to 6 nodes (pN2), and 8 patients had metastasis involving 7 or more nodes. The patients’ characteristics are shown in **Table 1**.

**Table 1:** Patient characteristics.

Characteristic	Ki67- Low expression (n=16)	Ki67-High expression (n=41)	P value
Age (years)	61.06 ± 10.53	62.12 ± 8.42	0.693
Sex			0.154
Female	1 (9.1%)	10 (90.9%)	
Male	15 (32.6%)	31 (67.4%)	
Tumor location			1.000
Upper	1 (25%)	3 (75%)	
Middle	7 (29.2%)	17 (70.8%)	
Lower	8 (27.6%)	21 (72.4%)	
Lymph node metastasis			1.000
Negative	9 (28.1%)	23 (71.9%)	
Positive	7 (28.0%)	18 (72.0%)	
Pathological T stage			0.366
I-II	8 (36.4%)	14 (63.6%)	
III-IV	8 (22.9%)	27 (77.1%)	
Histological differentiation			
Poor	2 (15.4%)	11 (84.6%)	0.313
Moderate or high	14 (31.8%)	30 (68.2%)	

### Interobserver agreement

The measurements made by the two radiologists were consistent, demonstrating strong interobserver agreement. All ICC values > 0.750. The ICC values of quantitative variables for inter-observer measurements ranged from 0.782 (95% CI: 0.616–0.875) to 0.893 (95% CI: 0.855–0.965).

### DECT and clinicopathological parameters in different Ki-67 statuses

**Figure 1:**
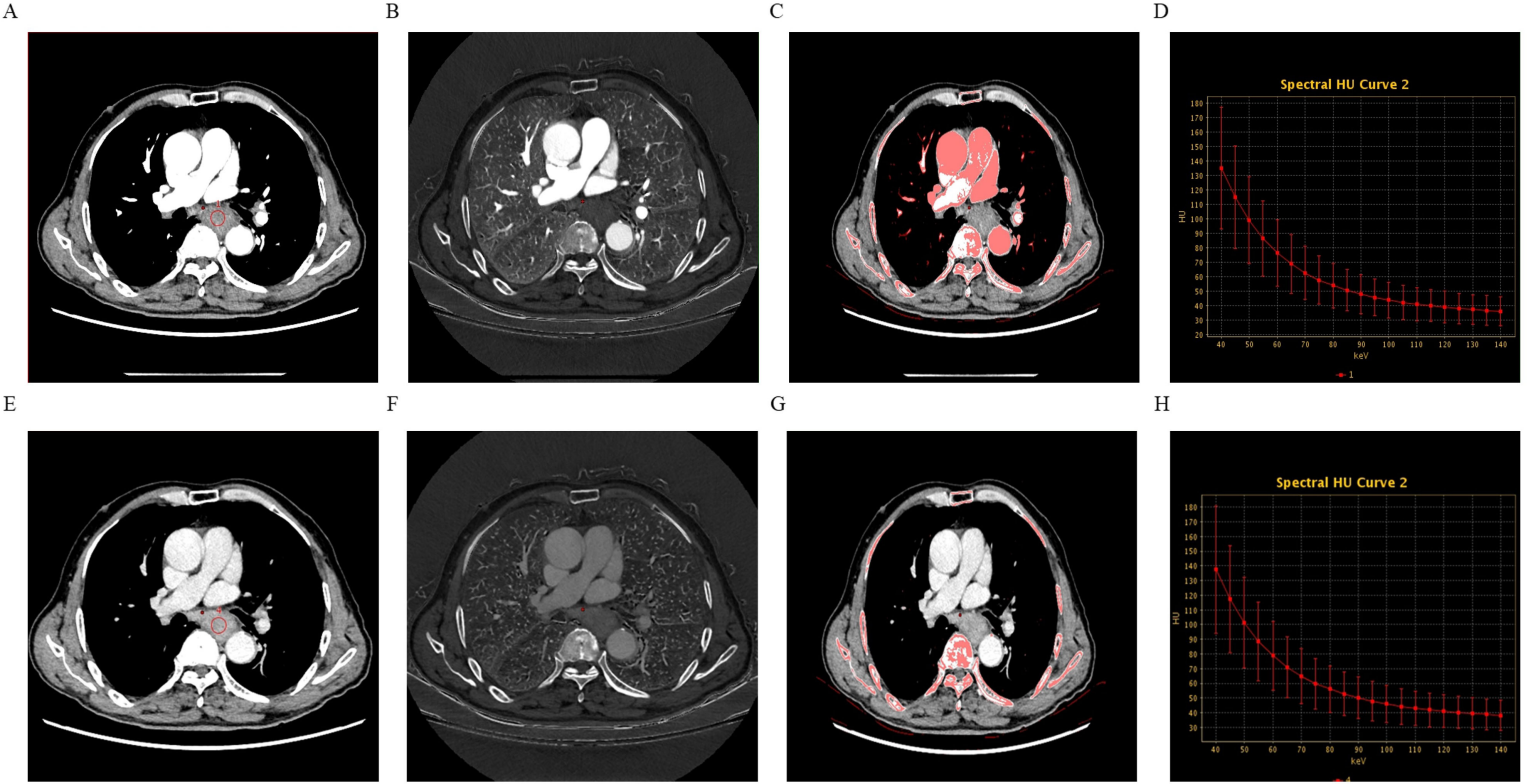
A 74-year-old male patient with esophageal squamous cell carcinoma (ESCC) exhibiting low Ki-67 expression, grade 2, without neurovascular invasion or lymph node metastasis. Panels **(A–D)** show the 70-keV monochromatic contrast-enhanced CT image, iodine-based map, effective atomic number (Zeff) map, and spectral curve derived from the arterial phase (AP) scans. Panels **(E–H)** display the same imaging parameters derived from the venous phase (VP) scans.

Univariate analysis demonstrated that, compared to the low-expression state of Ki-67, the high-expression state of Ki-67 was significantly associated with higher Zeff in the VP (ZeffVP) and IC in the VP (ICVP), as well as lower WC in the AP (WCAP) (P < 0.05). Although the high Ki-67 expression state exhibited slightly higher iodine concentration (ICAP) and effective atomic number (ZeffAP) in the AP, as well as slightly lower WC in the VP (WCVP) compared to the low Ki-67 expression state, these differences did not reach statistical significance. Similarly, there were no significant differences in the λHU and NIC values for the AP and VP phases between the two groups. Detail information is shown in **Table 2**. Representative DECT images of ESCC with low Ki-67 expression are shown in **Figure 1**.

**Table 2:** DECT parameters between low and high Ki-67 expression status.

Parameters	Ki67-Low expression (n=16)	Ki67-High expression (n=41)	P value	AUC (95% CI)	cut-off value	Sensitivity	Specificity
AP							
λHU	2.30 ± 0.91	2.50 ± 0.79	0.413				
IC (mg/mL)	12.21 ± 4.77	13.26 ± 4.15	0.412				
WC (mg/mL)	1029.76 (1027.53,1036.20)	1027.44 (1024.08,1030.24)	0.021	0.70 (0.55-0.85)	1028.520	0.659	0.688
Zeff	8.33 ± 0.28	8.40 ± 0.24	0.384				
NIC	0.11(0.10,0.15)	0.14 (0.12,0.20)	0.063				
VP							
λHU	3.55 (3.19, 4.11)	4.00 (3.65, 4.48)	0.050				
IC (mg/mL)	18.85 (16.88,21.76)	21.20 (19.38,24.52)	0.049	0.67 (0.51-0.82)	19.030	0.805	0.562
WC (mg/mL)	1034.95 ± 7.87	1032.91 ± 7.34	0.359				
Zeff	8.74 ± 0.17	8.87 ± 0.23	0.047	0.67 (0.52-0.82)	8.715	0.805	0.562
NIC	0.45 ± 0.09	0.47 ± 0.09	0.599				

Data are mean ± SD or median with interquartile range in parentheses. λHU, the slope of the spectral curve; IC, Iodine concentration; WC, Water concentrations; Zeff, Effective atomic number; NIC, Normalized iodine concentration; AP, Arterial phase; VP, Venous phase.

### Multivariate analysis of DECT parameters and Ki-67 expression

Following univariate analysis (P < 0.10), candidate variables were identified. Variables exhibiting multicollinearity (VIF ≥ 5) were subsequently excluded. Consequently, the final multivariable model retained only the most clinically meaningful and statistically significant predictors: ICVP and WCAP. The results showed that ICVP(odds ratio [OR]: 2.278; 95% confidence interval [CI]: 1.114–5.648) and WCAP(OR: 0.375; 95% CI: 0.147–0.775) were independent predictors of high Ki−67 expression in ESCC.​ The model constructed was as follow: Logit (P) = 1.215 + 0.823×ICVP−0.981×WCAP.

### Diagnostic value of DECT parameters for Ki-67 status

**Figure 2:**
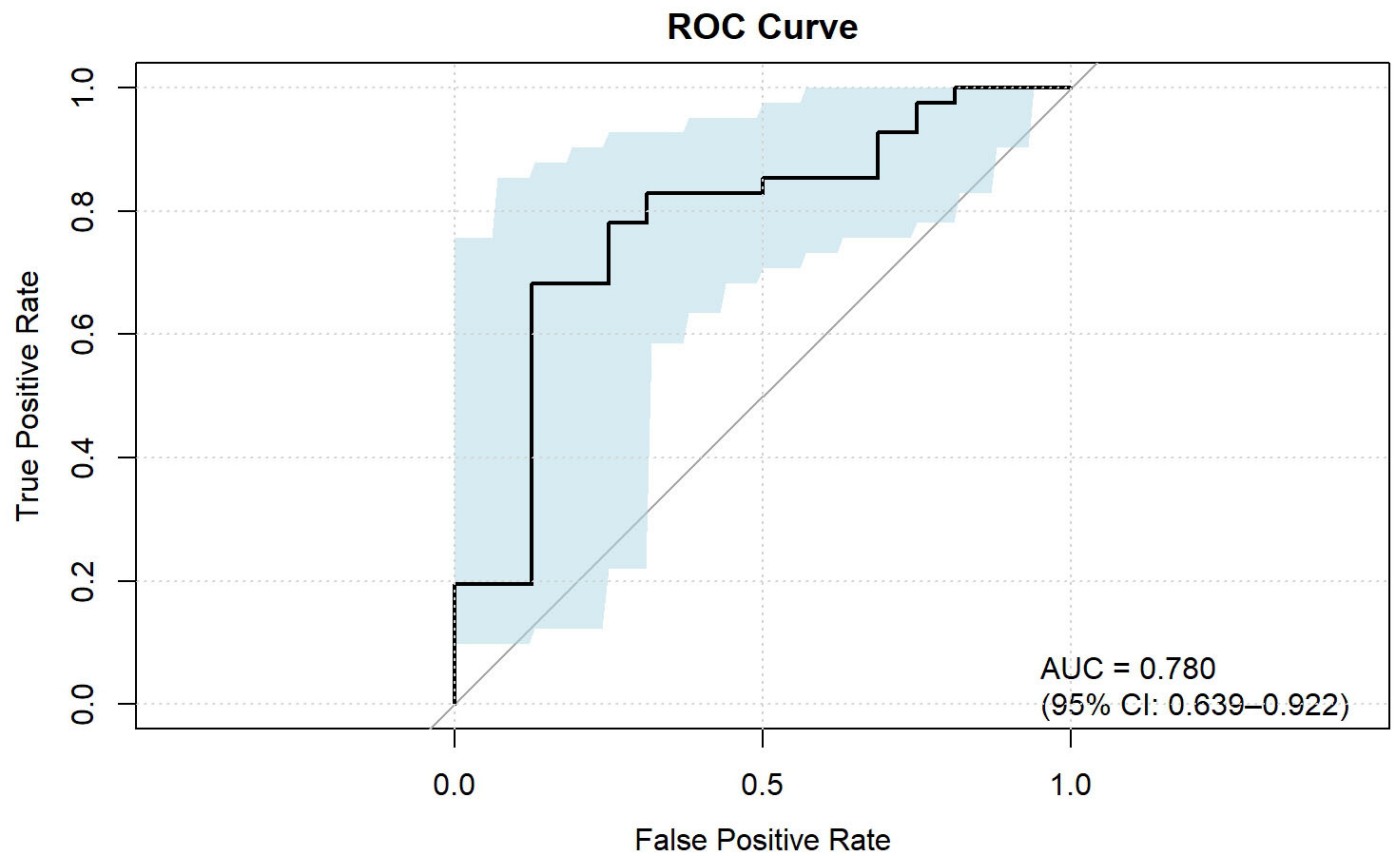
ROC curve of the combined parameters for predicting Ki-67 expression in ESCC. IC, Iodine concentration; WC, Water concentrations; Zeff, Effective atomic number; AP, Arterial phase; VP, Venous phase.

**Figure 3:**
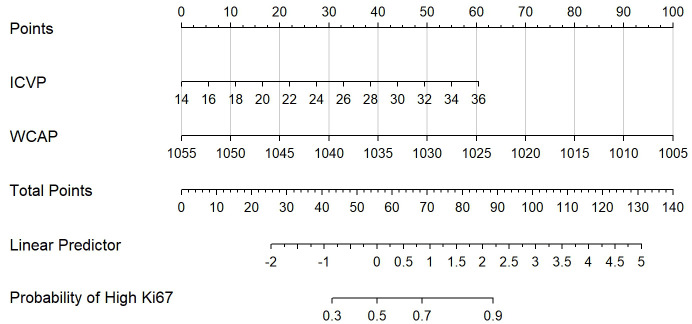
The Dual-energy CT (DECT) nomogram for evaluating the Ki-67 expression in ESCC. IC, Iodine concentration; WC, Water concentrations; AP, Arterial phase; VP, Venous phase.

**Figure 4:**
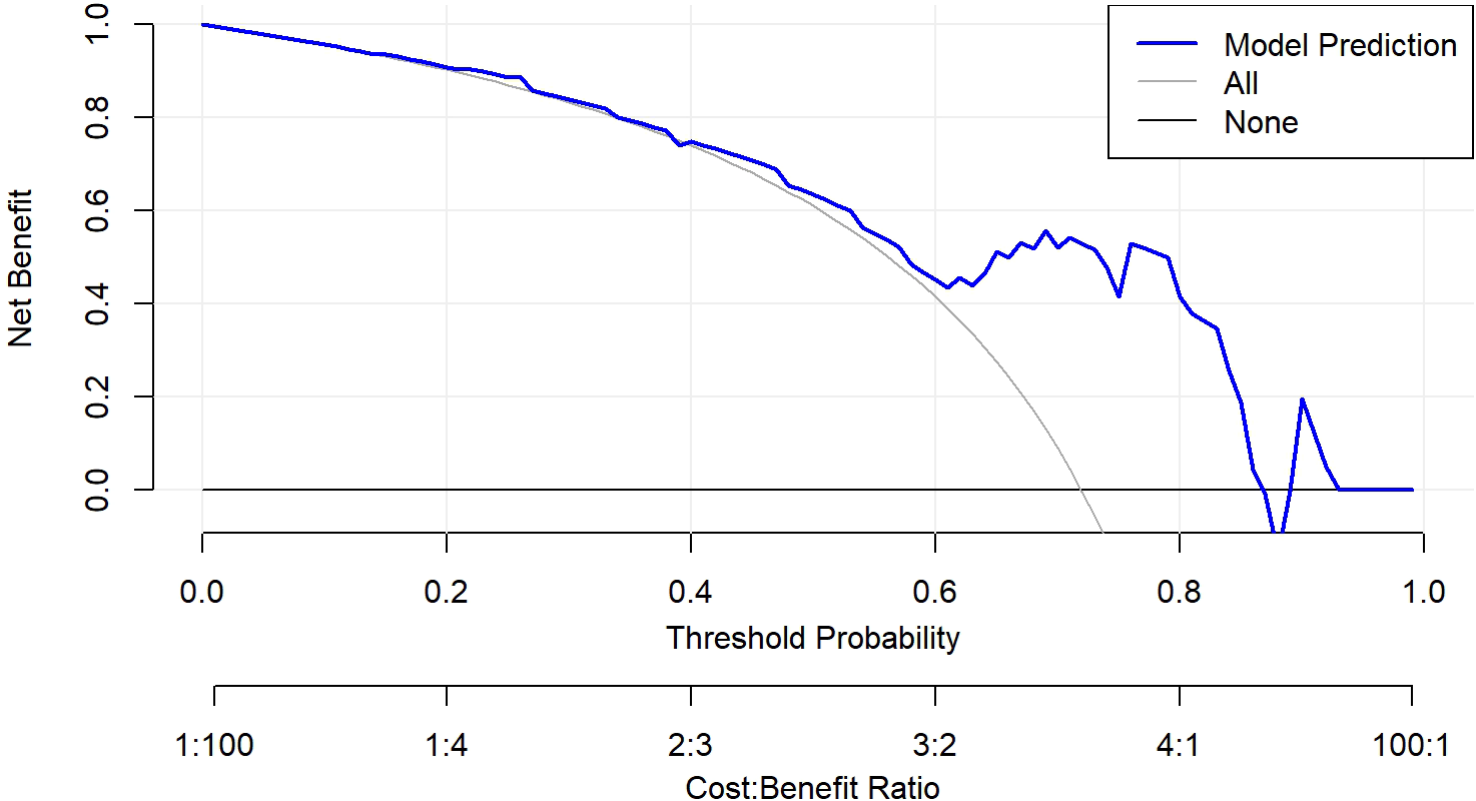
Decision curve analysis of the nomogram model. The nomogram demonstrated good calibration performance and provided the highest clinical net benefit across a wide range of threshold values, particularly demonstrating a positive net benefit within the threshold ranges of 0.12–0.99.

**Figure 5:**
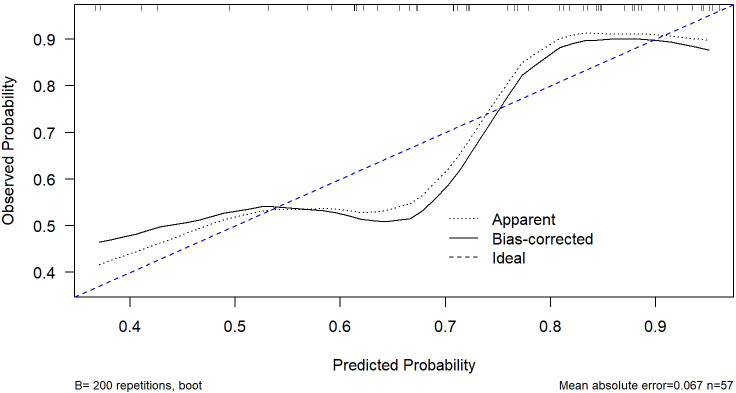
Calibration curve of the nomogram model. The curve was generated using bootstrap resampling (B = 200). The dashed blue line represents the ideal calibration line where predicted probabilities perfectly match observed outcomes. The Hosmer-Lemeshow test showed no significant lack of fit (p = 0.12), indicating good agreement between predicted and observed probabilities.

ROC analysis demonstrated that the optimal thresholds for single variable WCAP, ICVP, and ZeffVP in discriminating Ki-67 status were 1028.52, 19.03, and 8.72, with AUCs of 0.70, 0.67, and 0.67 (95% CI: 0.55–0.85, 0.51–0.82, and 0.52–0.82, respectively), sensitivities of 65.9%, 80.5%, and 80.5%, specificities of 68.8%, 56.2%, and 56.2%, and accuracies of 66.7%, 73.7%, and 73.7%, respectively. The combined WCAP + ICVP model for predicting Ki-67 status had an AUC of 0.78 (95% CI: 0.64–0.92; P < 0.001), with a cutoff value of 0.74, sensitivity of 68.3%, specificity of 87.5%, and accuracy of 73.7% (**Figure 2**). Subsequently, a DECT based nomogram was developed using the final factors and derived regression coefficients to predict the Ki67 expression status (**Figure 3**). To ensure the robustness and clinical relevance of our model despite the fixed cohort, we have conducted rigorous validations including Bootstrap resampling (1,000 iterations) showing AUC 95% CI of 0.64–0.92 with key indicators’ confidence intervals exceeding random levels, and sample adequacy verification indicating events per variable at 16:1 > 10:1 to meet logistic regression standards. The clinical value is assured by high specificity (87.5%, 95% CI: 0.81–0.79) to avoid over-treatment in low-risk patients. The decision curve analysis showed a net benefit across predicted probability thresholds ranging form0.12–0.99 (**Figure 4**). The Hosmer-Lemeshow test indicated good model calibration (p = 0.12). Together with the calibration curve generated by the bootstrap method, these results demonstrate that the predictive model performs stably and fits well in this dataset (**Figure 5**).

## Discussion

Ki-67, a well-established cellular proliferation marker, is widely utilized in both experimental and clinical contexts to assess tumor cell proliferation and predict patient prognosis. Elevated Ki-67 expression has been consistently associated with poor clinical outcomes in patients with EC, particularly ESCC. This pilot study aimed to explore the potential role of DECT-derived parameters in predicting Ki-67 status in ESCC patients.

In DECT imaging, iodine-water pairing in the material decomposition technique is considered the most stable strategy. Iodine contrast agents, which are the primary agents used for enhancing tumor vasculature during imaging, serve as key indicators of blood supply. Mao et al (19) observed a positive correlation between Ki-67 expression levels and NIC and IC in gastric cancer. They suggested that higher Ki-67 expression within tumors is indicative of increased tumor proliferation and growth activity, which is associated with enhanced blood supply, leading to elevated IC and NIC. Similarly, Fan et al (20) reported a positive correlation between Ki-67 expression levels and NIC in rectal cancer.

Our study found significant differences in IC between Ki67-High expression and Ki67-Low expression groups. Specifically, in the VP, both the Zeff and IC were higher in the Ki-67-positive group compared to the Ki-67-negative group. Conversely, in the AP, the WC was lower in the Ki-67-positive group. Previous research has demonstrated that higher iodine concentrations in DECT images correlate with increased tumor blood supply and a more aggressive tumor phenotype, with a greater likelihood of early recurrence in ESCC cases (21). The distribution of iodine in tissues is closely linked to local blood volume and vascular density, with elevated IC values reflecting greater blood supply to the tumor (22, 23). Some studies have suggested that NIC reduces the impact of circulatory variability on iodine content, providing a more accurate representation of the tumor’s blood supply (24). However, NIC can be influenced by factors such as the enhancement of the aorta, and for tumors with insufficient blood supply, NIC may not accurately reflect the true iodine concentration of the lesion (25, 26). Our findings suggest that IC, rather than NIC, may be a more reliable indicator of proliferative activity in tumors, aligning with previous reports on the utility of IC in assessing tumor blood supply and proliferation (23, 27).

Eff-Z, which indicates the atomic configuration of compounds or mixtures, is another key parameter in spectral CT that can differentiate tissues with similar attenuation properties (19, 28, 29). Our results showed significant differences in Eff-Z between the two groups, with Ki-67-high expression tumors exhibiting higher Eff-Z values. The increased Ki-67 expression is often associated with tumors possessing complex internal structures, high protein content, abnormal nuclear-to-cytoplasmic ratios, and compact extracellular spaces (30). This suggests that Eff-Z may reflect the structural and biochemical complexity of highly proliferative tumors.

Additionally, WCAP provides complementary information to IC, especially in VP, in DECT imaging. Previous studies have demonstrated that WC is less affected by the beam hardening effect, and can reliably depict the lesion characteristics for tumor differentiation (31–33).Our findings indicate that WCAP and ICVP, when used together, can provide robust evaluation metrics for Ki-67 expression in ESCC.

However, several limitations should be considered. First, this was a retrospective study with a relatively small sample size, which may affect the stability of the model. Nevertheless, we have ensured the reliability of the results within the constraints of the current sample size through rigorous internal validation: A 1,000-iteration Bootstrap resampling analysis showed that the model’s AUC had a 95% confidence interval (CI) of 0.64–0.92, with the original AUC value (0.78) located in the middle of the interval, indicating no significant bias. The lower bound of the interval (0.64) remained above the random level (0.5), confirming the model’s stable discriminative ability. Validation results for other key indicators also demonstrated good stability, including accuracy (73.7%, 95% CI: 0.68–0.79), sensitivity (68.3%, 95% CI: 0.61–0.75), specificity (87.5%, 95% CI: 0.81–0.94), and F1 score (78.9%, 95% CI: 0.73–0.84). Sample size adequacy assessment revealed that the ratio of high Ki-67 expression events to predictors was 16:1, significantly exceeding the 10:1 standard recommended for logistic regression, effectively reducing the risk of overfitting. Second, the study focused exclusively on ESCC, limiting its generalizability to other types of esophageal lesions. Future prospective studies with larger sample sizes and multi-center designs are warranted to further validate the model’s robustness and enhance its external validity. Additionally, expanding the scope to include a broader range of esophageal lesions (such as adenocarcinoma or pre-malignant lesions) would help clarify the generalizability of our findings across different pathological subtypes.

## Conclusion

In conclusion, DECT imaging parameters—particularly IC and Zeff in the VP, and WC in the AP—can provide valuable supplementary information for assessing Ki-67 status in ESCC. This approach may be particularly useful in clinical settings where biopsy or surgery is not feasible, offering a non-invasive method for prognosis and treatment planning.

## Data Availability

The raw data supporting the conclusions of this article will be made available by the authors, without undue reservation.

## References

[1] Zhu H Ma X Ye T Wang H Wang Z Liu Q. Esophageal cancer in China: Practice and research in the new era. Int J Cancer. (2023) 152:1741–51. doi: 10.1002/ijc.34301, PMID: 36151861

[2] Sung H Ferlay J Siegel RL Laversanne M Soerjomataram I Jemal A. Global cancer statistics 2020: GLOBOCAN estimates of incidence and mortality worldwide for 36 cancers in 185 countries. CA: Cancer J Clin. (2021) 71:209–49. doi: 10.3322/caac.21660, PMID: 33538338

[3] Weidenbaum C Gibson MK. Approach to localized squamous cell cancer of the esophagus. Curr Treat options Oncol. (2022) 23:1370–87. doi: 10.1007/s11864-022-01003-w, PMID: 36042147 PMC9526684

[4] Wang YK Karmakar R Mukundan A Men TC Tsao YM Lu SC. Computer-aided endoscopic diagnostic system modified with hyperspectral imaging for the classification of esophageal neoplasms. Front Oncol. (2024) 14:1423405. doi: 10.3389/fonc.2024.1423405, PMID: 39687890 PMC11646837

[5] Yang PC Huang CW Karmakar R Mukundan A Chen TH Chou CK. Precision imaging for early detection of esophageal cancer. Bioengineering (Basel Switzerland). (2025) 12. doi: 10.3390/bioengineering12010090, PMID: 39851364 PMC11762345

[6] He H Chen N Hou Y Wang Z Zhang Y Zhang G. Trends in the incidence and survival of patients with esophageal cancer: A SEER database analysis. Thorac Cancer. (2020) 11:1121–8. doi: 10.1111/1759-7714.13311, PMID: 32154652 PMC7180574

[7] Du M Wang X Zhuang S Lou K Li G Xie X. Quantitative parameters in novel spectral computed tomography for assessing gastric cancer and cell proliferation. Eur J Radiol. (2023) 167:111052. doi: 10.1016/j.ejrad.2023.111052, PMID: 37643557

[8] Zheng J Huang B Chen Y Zeng B Xiao L Wu M. Exploratory analyses of the associations between Ki-67 expression, lymph node metastasis, and prognosis in patients with esophageal squamous cell cancer. PeerJ. (2025) 13:e19062. doi: 10.7717/peerj.19062, PMID: 40028218 PMC11871893

[9] Wang Y Dai M Chen X. Prognostic and clinicopathological value of Ki-67 in patients with oesophageal squamous cell carcinoma: a systematic review and meta-analysis. BMJ Open. (2024) 14:e083637. doi: 10.1136/bmjopen-2023-083637, PMID: 38839387 PMC11163609

[10] Zhang X Chen Y Li Z Han X Liang Y. TGFBR3 is an independent unfavourable prognostic marker in oesophageal squamous cell cancer and is positively correlated with Ki-67. Int J Exp Pathol. (2020) 101:223–9. doi: 10.1111/iep.12380, PMID: 33146446 PMC7691215

[11] Rimm DL Leung SCY McShane LM Bai Y Bane AL Bartlett JMS. An international multicenter study to evaluate reproducibility of automated scoring for assessment of Ki67 in breast cancer. Modern Pathol. (2019) 32:59–69. doi: 10.1038/s41379-018-0109-4, PMID: 30143750

[12] Wang P Tang Z Xiao Z Wu L Hong R Duan F. Dual-energy CT in predicting Ki-67 expression in laryngeal squamous cell carcinoma. Eur J Radiol. (2021) 140:109774. doi: 10.1016/j.ejrad.2021.109774, PMID: 34004427

[13] Liu W Xie T Chen L Tang W Zhang Z Wang Y. Dual-layer spectral detector CT: A noninvasive preoperative tool for predicting histopathological differentiation in pancreatic ductal adenocarcinoma. Eur J Radiol. (2024) 173:111327. doi: 10.1016/j.ejrad.2024.111327, PMID: 38330535

[14] Tian S Jianguo X Tian W Li Y Hu J Wang M. Application of dual-energy computed tomography in preoperative evaluation of Ki-67 expression levels in solid non-small cell lung cancer. Medicine. (2022) 101:e29444. doi: 10.1097/md.0000000000029444, PMID: 35945799 PMC9351836

[15] Wu Y Li J Ding L Huang J Chen M Li X. Differentiation of pathological subtypes and Ki-67 and TTF-1 expression by dual-energy CT (DECT) volumetric quantitative analysis in non-small cell lung cancer. Cancer imaging: Off Publ Int Cancer Imaging Soc. (2024) 24:146. doi: 10.1186/s40644-024-00793-6, PMID: 39456114 PMC11515807

[16] Wen Y Song Z Li Q Zhang D Li X Yu J. Development and validation of a model for predicting the expression of Ki-67 in pancreatic ductal adenocarcinoma with radiological features and dual-energy computed tomography quantitative parameters. Insights Imaging. (2024) 15:41. doi: 10.1186/s13244-024-01617-8, PMID: 38353857 PMC10866831

[17] Li SH Chen CH Lu HI Huang WT Tien WY Lan YC. Phosphorylated p70S6K expression is an independent prognosticator for patients with esophageal squamous cell carcinoma. Surgery. (2015) 157:570–80. doi: 10.1016/j.surg.2014.10.014, PMID: 25726316

[18] Lim CH Park YJ Shin M Cho YS Choi JY Lee KH. Tumor SUVs on 18F-FDG PET/CT and aggressive pathological features in esophageal squamous cell carcinoma. Clin Nucl Med. (2020) 45:e128–33. doi: 10.1097/rlu.0000000000002926, PMID: 31977480

[19] Mao LT Chen WC Lu JY Zhang HL Ye YS Zhang Y. Quantitative parameters in novel spectral computed tomography: Assessment of Ki-67 expression in patients with gastric adenocarcinoma. World J Gastroenterol. (2023) 29:1602–13. doi: 10.3748/wjg.v29.i10.1602, PMID: 36970586 PMC10037253

[20] Fan S Li X Zheng L Hu D Ren X Ye Z. Correlations between the iodine concentrations from dual energy computed tomography and molecular markers Ki-67 and HIF-1α in rectal cancer: A preliminary study. Eur J Radiol. (2017) 96:109–14. doi: 10.1016/j.ejrad.2017.08.026, PMID: 29103468

[21] Liu Y Cheng F Wang L Du L Shen H Wang X. Quantitative parameters derived from dual-energy computed tomography for the preoperative prediction of early recurrence in patients with esophageal squamous cell carcinoma. Eur Radiol. (2023) 33:7419–28. doi: 10.1007/s00330-023-09818-3, PMID: 37314470

[22] Wang X Liu D Zeng X Jiang S Li L Yu T. Dual-energy CT quantitative parameters for evaluating Immunohistochemical biomarkers of invasive breast cancer. Cancer Imaging. (2021) 21:4. doi: 10.1186/s40644-020-00370-7, PMID: 33413654 PMC7791709

[23] Li Q Li X Li XY Huo JW Lv FJ Luo TY. Spectral CT in lung cancer: usefulness of iodine concentration for evaluation of tumor angiogenesis and prognosis. AJR Am J roentgenol. (2020) 215:595–602. doi: 10.2214/ajr.19.22688, PMID: 32569515

[24] Zhan Y Wang Y Wang P Wang Y Ni X Wang J. Pretreatment dual-energy CT for predicting early response to induction chemotherapy and survival in nasopharyngeal carcinoma. Eur Radiol. (2023) 33:9052–62. doi: 10.1007/s00330-023-09837-0, PMID: 37405505

[25] Chen XH Ren K Liang P Chai YR Chen KS Gao JB. Spectral computed tomography in advanced gastric cancer: Can iodine concentration non-invasively assess angiogenesis? World J Gastroenterol. (2017) 23:1666–75. doi: 10.3748/wjg.v23.i9.1666, PMID: 28321168 PMC5340819

[26] Pang LF Zhang H Lu W Yang WJ Xiao H Xu WQ. Spectral CT imaging of myocardial infarction: preliminary animal experience. Eur Radiol. (2013) 23:133–8. doi: 10.1007/s00330-012-2560-9, PMID: 22814826

[27] Zhang Z Zou H Yuan A Jiang F Zhao B Liu Y. A single enhanced dual-energy CT scan may distinguish lung squamous cell carcinoma from adenocarcinoma during the venous phase. Acad Radiol. (2020) 27:624–9. doi: 10.1016/j.acra.2019.07.018, PMID: 31447258

[28] Li WX Miao F Xu XQ Zhang J Wu ZY Chen KM. Pancreatic neuroendocrine neoplasms: CT spectral imaging in grading. Acad Radiol. (2021) 28:208–16. doi: 10.1016/j.acra.2020.01.033, PMID: 32111466

[29] Mileto A Allen BC Pietryga JA Farjat AE Zarzour JG Bellini D. Characterization of incidental renal mass with dual-energy CT: diagnostic accuracy of effective atomic number maps for discriminating nonenhancing cysts from enhancing masses. AJR Am J roentgenol. (2017) 209:W221–w230. doi: 10.2214/ajr.16.17325, PMID: 28705069

[30] Lin L Cheng J Tang D Zhang Y Zhang F Xu J. The associations among quantitative spectral CT parameters, Ki-67 expression levels and EGFR mutation status in NSCLC. Sci Rep. (2020) 10:3436. doi: 10.1038/s41598-020-60445-0, PMID: 32103127 PMC7044288

[31] Zhang B Wu Q Qiu X Ding X Wang J Li J. Effect of spectral CT on tumor microvascular angiogenesis in renal cell carcinoma. BMC Cancer. (2021) 21:874. doi: 10.1186/s12885-021-08586-x, PMID: 34330234 PMC8325217

[32] Elsherif SB Zheng S Ganeshan D Iyer R Wei W Bhosale PR. Does dual-energy CT differentiate benign and Malignant ovarian tumours? Clin Radiol. (2020) 75:606–14. doi: 10.1016/j.crad.2020.03.006, PMID: 32252992

[33] Liu G Li M Li G Li Z Liu A Pu R. Assessing the blood supply status of the focal ground-glass opacity in lungs using spectral computed tomography. Korean J Radiol. (2018) 19:130–8. doi: 10.3348/kjr.2018.19.1.130, PMID: 29354009 PMC5768493

